# Post-transcriptional inhibition of hepatitis C virus replication through small interference RNA

**DOI:** 10.1186/1743-422X-8-112

**Published:** 2011-03-10

**Authors:** Usman Ali Ashfaq, Muhammad Ansar, Muhammad Tahir Sarwar, Tariq Javed, Sidra Rehman, Sheikh Riazuddin

**Affiliations:** 1 Division of Molecular Medicine, National Centre of Excellence in Molecular Biology, University of the Punjab, Lahore, Pakistan; 2 Allama Iqbal Medical College, Allama Shabir Ahmad Usmani Road, Lahore, Pakistan

## Abstract

**Background:**

Hepatitis C Virus (HCV) infection is a major health problem throughout world that causes acute and chronic infection which resulted in liver fibrosis, hepatocellular carcinoma and death. The only therapy currently available for HCV infection is the combination of pegylated interferon alpha (PEG-IFN α) and ribavirin. This therapy can effectively clear the virus infection in only 50% of infected individuals. Hence, there is a dire need to develop antiviral agents against HCV.

**Results:**

This study was design to examine the ability of exogenous small interfering RNAs (siRNAs) to block the replication of HCV in human liver cells. In the present study six 21-bp siRNAs were designed against different regions of HCV non-structural genes (NS2, NS3 serine protease/helicase, NS4Band NS5B RNA dependent RNA polymerase). siRNAs were labeled as NS2si241, NS3si-229, NS3si-858, NS4Bsi-166, NS5Bsi-241 and NS5Bsi-1064. We found that siRNAs against HCV NS2- NS5B efficiently inhibit HCV replication in Huh-7 cells. Our results demonstrated that siRNAs directed against HCV NS3 (NS3si-229 and NS3si-858) showed 58% and 88% reduction in viral titer respectively. Moreover, NS4Bsi-166 and NS5Bsi-1064 exhibited a dramatic reduction in HCV viral RNA and resulted in greater than 90% inhibition at a 20 μM concentration, while NS2si-241 showed 27% reduction in viral titer. No significant inhibition was detected in cells transfected with the negative control siRNA.

**Conclusion:**

Our results suggest that siRNAs targeting against HCV non-structural genes (NS2-NS5B) efficiently inhibit HCV replication and combination of these siRNAs of different targets and interferon will be better option to treat HCV infection throughout the world.

## Background

HCV has infected 200 million people worldwide, of which 10 million individuals (6% of the population) have been spotted in Pakistan [1]. In 40-60% of HCV-infected individuals, persistent infection is mainly associated with liver cirrhosis and steatosis, leading to hepatocellular carcinoma (HCC) [2,3]. About 75% of patients receive no therapeutic benefit from the current combination therapy with PEG-IFN α and the guanosine analog ribavirin because of adverse side effects and high cost [4]. In order to improve treatment outcomes, there is a dire need to develop more effective and better therapeutic options for treating HCV infections.

Currently, RNA interference (RNAi) has been emerged as a potential technique for developing anti-mRNA based therapeutics against different viral diseases such as HPV [5], HIV [6], Yellow fever virus [7,8]. RNAi is a sequence-specific RNA degradation process in the cytoplasm of eukaryotic cells triggered by double-stranded RNA (dsRNA), widely existing in many species from nematode to human [Fire, 1998, Elbashir, 2001, Leung, 2005,]. Upon introduction into the cells, exogenous dsRNAs are cut into 21-25 nt small interfering RNA (siRNA) by an RNase III-like enzyme called Dicer. The siRNAs form RNA-induced silencing complex (RISC) with other cellular components, and lead to the cleavage of their homologous transcript and eventually the silencing of specific gene [9-11]. HCV RNA is an attractive target for RNAi, as the single positive-stranded viral transcript functions both as genomic RNA and a replication template, and also because of its localization in the infected liver, an organ that can be readily targeted by nucleic acid molecules and viral vectors. As Dicer and the RISC act in the cytoplasm so the cytoplasmic location of RNAi machinery makes it technically easier than other methods that attempt to silence genes at the nuclear level. Several reports demonstrated that siRNAs against HCV genomic and sub-genomic replicons inhibit HCV replication [12-16].

HCV was firstly recognized in 1989 [17], comprising a 9.6 kb genome of positive sense. It encodes a single large polyprotein of 3010 amino acids is translated from the long open reading frame (ORF) encoded within the viral RNA genome. This large protein is then cleaved into 10 different individual proteins by the combined action of the cellular and viral proteases. The viral core, E1, E2, and P-7 proteins are called the structural proteins required for the production of infectious virus particles, their secretion and infection. The remaining non-structural proteins (NS2, NS3, NS4A, NS4B, NS5A, NS5B) are essential for replication of HCV positive and negative strand RNA [18]. Among these non-structural proteins, HCV NS3 serine protease and NS5b RNA dependent RNA Polymerase are important targets to develop antiviral drugs against HCV [19,20]. The present study was devise to study the effect of siRNAs against HCV replication in liver infected cells. The present study demonstrates that the RNAi-mediated silencing of the HCV full length viral particle may be one of the important therapeutic opportunities against HCV 1a genotype.

## Material methods

### Serum Sample Collection

HCV-1a patient's serum samples used in this investigation were obtained from the CAMB (Center for Applied Molecular Biology) diagnostic laboratory, Lahore, Pakistan. Serum samples were stored at -80°C prior to viral inoculation experiments. Quantification and genotype was assessed by CAMB diagnostic laboratory, Lahore, Pakistan. Patient's written consent and approval for this study was obtained from institutional ethics committee.

### siRNA designing

Small interfering RNA oligonucleotides against HCV non structural genes (NS2-NS5B) were designed to the most conserved target region of these genes using the Ambion's siRNA design tool http://www.ambion.com/techlib/misc/siRNA_finder.html. The designed siRNAs against HCV non structural genes were synthesized using Silencer siRNA construction kit according to the manufacturer's instruction (Ambion, USA).

### Cell line

The Huh-7 cell line was compassionately offered by Dr. Zafar Nawaz (Biochemistry and Molecular Biology Department, University of Miami, USA). Huh-7 cells were cultured in Dulbecco's modified Eagle medium (DMEM) supplemented with 10% fetal bovine serum & 100 IU/ml penicillin & 100 μg/ml streptomycin, at 37°C in an atmosphere of 5% CO_2_.

### Anti-HCV analysis of siRNAs on Huh-7 cells

Huh-7 cell line was used to establish the in-vitro replication of HCV. A similar protocol was used for viral inoculation as established by Zekari et al. 2009 [21] and El-Awardy et al. 2006 [22]. High viral titer >1 × 10^8 ^IU/ml from HCV-1a patient's was used as principle inoculum in these experiments. Huh-7 cells were maintained in 6-well culture plates to semi-confluence, washed twice with serum-free medium, then inoculated with 500 μl (5 × 10^7^IU/well) and 500 μl serum free media. Cells were maintained overnight at 37°C in 5% CO_2_. Next day, adherent cells were washed three times with 1 × PBS, complete medium was added and incubation was continued for 48 h. Cells were harvested and assessed for viral RNA quantification by Real Time PCR. To analyze the effect of siRNAs on HCV infection, serum infected Huh-7 cells were again seeded after three days of infection in 24-well plates in the presence and absence of siRNAs and grown to 80% confluence. After 72 h, total RNA was isolated by using Gentra RNA isolation kit (Gentra System Pennsylvania, USA) according to the manufacturer's instructions. Briefely, cells were lysed with cell lysis solution containing 5 μl internal control (Sacace Biotechnologies Caserta, Italy). RNA pallet was solubilized in 1% DEPC (Diethyl pyrocarbonate treated water). HCV RNA quantifications were determined by Real Time PCR Smart Cycler II system (Cepheid Sunnyvale, USA) using the Sacace HCV quantitative analysis kit (Sacace Biotechnologies Caserta, Italy) according to the manufacturer's instructions.

### Formula for the calculation of HCV RNA concentration

Following formula was used to calculate the concentration HCV RNA of each sample.

IC = internal control, which is specific for each lot.

### Statistical Analysis

All statistical analysis was done using SPSS software (version 11.0, SPSS Inc). Data is presented as mean + SE. Numerical data was analyzed using student's t-test and ANOVA. P value < 0.05 was considered statistically significant.

## Results

**Figure 1:**
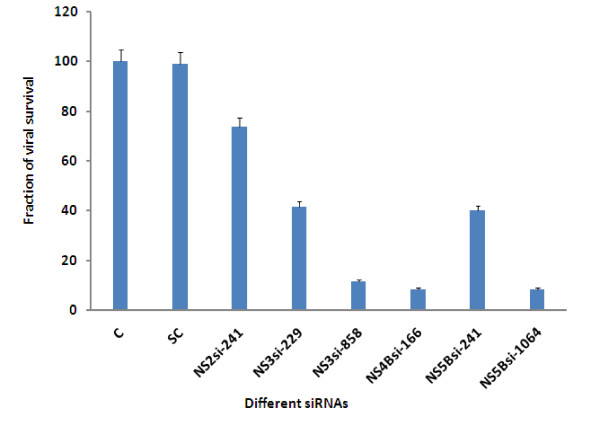
**Antiviral effect of siRNAs against HCV viral replication**. Huh-7 cells were infected with high titer sera sample from HCV-1a patients to establish in vitro cell culture model of HCV-1a, cells were maintained overnight at 37°C in 5% CO2 for three days. Cells were harvested after siRNA treatment 48 hrs post transfection and intracellular HCV RNA levels were quantified by Real Time PCR. Data is expressed as percentage of HCV survival in cells. Error bars indicate, mean S.D p < 0.05 verses control.

The ability of siRNAs to inhibit HCV replication were evaluated by designing and constructing siRNAs against different sites of HCV non-structural genes having genotype 1a (NS2, NS3, NS4B and NS5B). siRNA targeting sites were selected in regions conserved among different samples. Selected siRNAs were labeled as NS2si-241, NS3si-229, NS3si-858, NS4Bsi-166, NS5Bsi-241 and NS5Bsi-1065. The negative control siRNA was also included which has the same nucleotide composition as the experimental siRNA, but the sequence has been scrambled so that it does not has the significant sequence homology to with the HCV genes. The DNA olio templates were ordered from Sigma (Sigma Aldrich U.S.A.) (Table 1). We speculated that siRNAs design against HCV non-structural genes has the ability to inhibit the replication of HCV. To test this possibility, Huh-7 serum infected cells were treated with synthetic siRNAs and subsequently incubated for 3 days. Real time results showed that siRNAs targeted against non- structural genes inhibit HCV replication different from each other. siRNAs directed against HCV NS3 serine protease/helicase (NS3si-229 and NS3si-858) resulted in 58% and 82% reduction in viral titer, while siRNA against HCV NS2 gene (NS2si-241) showed 27% reduction in viral titer. siRNAs aginst HCV NS5B RNA dependent RNA polymerase and NS4B genes showed a dramatic reduction in HCV viral RNA, NS4Bsi-166 and NS5Bsi-1064 exhibited maximum inhibition up to 90% at a 20 μM concentration. No significant inhibition was detected in cells transfected with the negative control siRNA (Figure 1). This result was in accordance with Zekri et al. 2009 [21] who also showed best inhibitory effect of siRNAs against 5'UTR on 3rd day of post-transfection. Together, these data suggest that siRNA targeted against HCV non-structural genes inhibit HCV repliction.

**Table 1:** Sequence of siRNA oligonucleotides directed against HCV 1a non-structural genes

SN	siRNAs Name	Sequence 5'-3'
1	NS2si 241-antisense	AAACTACTCCTGGCCATCTTCCCTGTCTC
2	NS2si 241-sense	AAGAAGATGGCCAGGAGTAGTCCTGTCTC
3	NS3si 229-antisense	AATGTGGACCAAGACCTTGTGCCTGTCTC
4	NS3si 229-sense	AACACAAGGTCTTGGTCCACACCTGTCTC
5	NS3si 858-antisense	AATAATTTGTGACGAGTGCCACCTGTCTC
6	NS3si 858-sense	AATGGCACTCGTCACAAATTACCTGTCTC
7	NS4Bsi 166-antisense	AATTTCATCAGTGGGATACAACCTGTCTC
8	NS4Bsi 166-sense	AATTGTATCCCACTGATGAAACCTGTCTC
9	NS5Bsi 241-antisense	AACTTGCTATCCGTAGAGGAACCTGTCTC
10	NS5Bsi 241-sense	AATTCCTCTACGGATAGCAAGCCTGTCTC
11	NS5Bsi 1064-antisense	AACCAGAATACGACTTGGAGCCCTGTCTC
12	NS5Bsi 1064-sense	AAGCTCCAAGTCGTATTCTGGCCTGTCTC

## Discussion

With the progress in advanced technologies, RNAi has not only become a powerful tool for studying gene function and development of gene-based therapies, but also been widely used to inhibit viral replication of viruses such as HIV [6], HBV [12], HCV [12], respiratory viruses [23]. Previous reports showed that, at the molecular, cellular and individual levels, RNAi can potentially be used to block viral transmission and thus prevent the viral diseases [24]. With the high efficiency, specificity and low cytotoxicity, RNAi offered a new promise of anti-viral therapy.

Huh-7 and its derived cell lines are the most widely used cell culture systems for liver-associated diseases and the development of antiviral agents against HCV [25-27]. Guha et al. reported that in-vitro cell culture models can demonstrate the infectivity of the virus and can be used for evaluating drugs for antiviral activity or inhibition of HCV infection [28]. Recently different groups have studied the HCV replication in serum infected liver cell lines which mimics the naturally occurring HCV virions biology and kinetics of HCV infection in human. We infected Huh-7 cells with native viral particles from HCV-1a positive serum using the same protocol as describe by El-Awady et al., and Zekri et al [21,22].

Previous reports have demonstrated that NS3 and NS5B is essential for viral replication and therefore, provides an attractive target for development of antiviral agents [29-31]. In this study, we showed that siRNAs can be used as a potent approach to reduce HCV replication in a sequence-specific manner. We screened six siRNAs targeting conserved region of HCV non- structural genes and examined their effect on viral replication. An exciting finding of this study is decline of HCV viral titer to a maximum of 90% with a gene specific siRNAs directed against HCV NS5B RNA dependent RNA polymerase and NS4B. HCV replication in the Huh-7 cells was observed through detection of 5'UTR of viral copies by Real Time PCR in cells 3rd day post infection. HCV NS3si-229 and NS5Bsi-241 showed greater than 50% inhibition in viral titer, while NS2si-241 exhibited only 27% reduction in viral titer. Other investigators have also reported the inhibition of HCV replication by targeting NS5A, NS3, or NS5B sequences [13,15,32,33]. Our data suggest that effect of siRNAs against HCV non- structural genes on HCV viral titer reduction is possibly due to the simultaneous degradation of HCV genomic RNA (as HCV genome contains a positive sense ssRNA).

In summary, these results clearly show that the siRNAs mediated viral gene silencing is a very effective antiviral strategy that has a very strong potential for curing chronic hepatitis C virus infection.

## Abbreviations

**HCV**: Hepatitis C virus; **PEG-INF**: Pegylated interferon; **siRNA**: Small interference RNA; **Huh-7**: Human Hepatoma Cell line.

## Competing interests

The authors declare that they have no competing interests.

## Authors' contributions

UAA, MA, MTS, TJ and SDR contributed equally in lab work and manuscript write up. SRD was the principal investigator and provides all facilitates to complete this work. All the authors read and approved the final manuscript.

## Authors' information

Usman Ali Ashfaq (PhD Molecular Biology), Tariq Javed (M.Phil pharmaceutical chemistry, Sidra Rehman (MSc Chemistry) and Sheikh Riazuddin (PhD molecular Biology and Dean Post graduate study at Allama Iqbal medical college, Lahore
